# Rapid Detection of Equine Piroplasms Using Multiplex PCR and First Genetic Characterization of *Theileria haneyi* in Egypt

**DOI:** 10.3390/pathogens10111414

**Published:** 2021-10-31

**Authors:** Bassma S. M. Elsawy, Ahmed M. Nassar, Heba F. Alzan, Raksha V. Bhoora, Sezayi Ozubek, Mona S. Mahmoud, Omnia M. Kandil, Olfat A. Mahdy

**Affiliations:** 1 Parasitology and Animal Diseases Department, Veterinary Research Institute, National Research Centre, Dokki, Giza 12622, Egypt; Bs.el-sawy@nrc.sci.eg (B.S.M.E.); Monasaid3000@yahoo.com (M.S.M.); kandil_om@yahoo.com (O.M.K.); 2 Tick and Tick-Borne Diseases Research Unit, Veterinary Research Institute, National Research Centre, Dokki, Giza 12622, Egypt; 3 Parasitology Department, Faculty of Veterinary Medicine, Cairo University, Giza 12622, Egypt; drahmednassar22@yahoo.com; 4 Department of Veterinary Microbiology and Pathology, College of Veterinary Medicine, Washington State University, Pullman, WA 99164, USA; sezayi.ozubek@wsu.edu; 5 Department of Veterinary Tropical Diseases, Faculty of Veterinary Science, University of Pretoria, Private Bag X04, Onderstepoort 0110, South Africa; raksha.vasantraibhoora@up.ac.za; 6 Department of Parasitology, Faculty of Veterinary Medicine, University of Firat, Elazig 23119, Turkey

**Keywords:** equids, *Babesia caballi*, *Theileria equi*, *Theileria haneyi*, multiplex PCR, cPCR, 18S rRNA gene and sequencing

## Abstract

Equine Piroplasmosis (EP) is an infectious disease caused by the hemoprotozoan parasites *Theileria equi*, *Babesia caballi*, and the recently identified species *T. haneyi*. Hereby, we used a multiplex PCR (mPCR) targeting the 18S rRNA gene of *T. equi* and *B. caballi* for the simultaneous detection of EP in Egyptian equids and examined the presence of *T. haneyi* infections in Egypt. Blood samples from 155 equids (79 horses and 76 donkeys) collected from different governorates of Egypt were examined by mPCR and PCR targeting *T. hayeni*. The mPCR method revealed a prevalence of *T. equi* of 20.3% in horses and of 13.1% in donkeys and a prevalence of *B*. *caballi* of 1.2% in horses. *B*. *caballi* was not detected in donkeys in the current study. The mPCR method also detected coinfections with both species (2.5% and 1.3% in horses and donkeys, respectively). Additionally, we report the presence of *T. haneyi* in Egypt for the first time in 53.1% of the horse and 38.1% of the donkey tested samples. Coinfection with *T. haneyi* and *T. equi* was found in 13.5% of the samples, while infection with the three EP species was found in 1.9% of the samples.

## 1. Introduction

In rural areas of many developing countries, including Egypt, there is a huge reliance on working equids, which include horses, donkeys, mules, and ponies. These animals play important roles in sustaining the livelihoods of millions of people by providing support in industries that include agriculture, construction, tourism, mining, and public transport [1,2]. The health and welfare of domesticated equids are often overlooked in rural areas. Although equids can be affected by a myriad of diseases that include amongst others, African Horse Sickness, Epizootic Lymphangitis (EZL), Tetanus, Rabies, Trypanosomiasis, and Piroplasmosis, there is a general lack of knowledge regarding the identification, management, and prevention of infectious diseases [3].

Equine piroplasmosis [EP] is one of the infectious tick-borne diseases (TBDs) of equids, caused by the hemoprotozoan parasites *T. equi, B. caballi* [4], and the newly identified species *T. haneyi* [5]. There are over 30 species of ticks that have been described as vectors of *T. equi*, *B. caballi*, or both, including the genera *Hyalomma*, *Rhipicephalus, Dermacentor*, *Amblyomma,* and *Haemaphysalis* [6]. In Egypt, three species of ticks have been detected in equids, i.e., *Hyalomma dromedarii*, *Hyalomma excavatum*, and *Rhipicephalus annulatus* [7]. However, investigations on vector competence for *T. haneyi* has not been reported [8]. Infection with *T. equi* and *B. caballi* can cause severe economic losses in the equine industry due to the cost of treatment, especially for acutely infected horses, which, in the absence of appropriate treatment, can die [2]. It was found that *T. haneyi* rarely causes clinical signs in field horses [9], even in splenectomized horses experimentally infected with *T. haneyi* using the intravenous (IV) route [5,10]. Horses infected with *T. equi* remain persistently infected, while those affected by *B. caballi* are infected for an extended period [11].

Phylogenetic analysis of published *T. equi* and *B. caballi* 18S rRNA (SSU rRNA) gene sequences have led to the identification of five *T. equi* genotypes (A, B, C, D, and E) and two *B. caballi* genotypes (A, B) globally. The genotype B of *B. caballi* was reclassified into two subgenotypes called genotype B1 and genotype B2 [12,13], but genotype B2 was renamed as genotype C [14,15,16]. *Theileria haneyi* was first detected in a horse at the U.S.– Mexico border, near Eagle Pass, Texas [5,10]. Phylogenetic evidence places this species into a clade distinct from that of *T. equi* [5], and this species also lacks the equi merozoite antigen 1 (*ema-1*) gene that occurs in *T. equi*, explaining the failure of a diagnostic cELISA test based on recombinant *T. equi* ema-1 antigen in detecting *T. haneyi* [9].

The control and treatment of EP in non-endemic countries depend mainly on serological and molecular techniques [17]. In Egypt, the national tick control program recommends the use of acaricides like doramectin to reduce tick exposure [18,19]. The diagnosis of EP based solely on clinical signs is not specific, and differentiation between the EP causative agents is not possible based on clinical signs alone [20]. Microscopical examination (ME) of blood films has limited utility due to its low sensitivity, particularly in carrier animals with low parasitemia [21]. In addition, these diagnostic tools cannot identify and genetically characterize species of *Babesia* and *Theileria* infecting equids. In Egypt, the diagnosis of EP is based on ME and/or small-scale surveys using conventional PCR (cPCR) for the diagnosis of each species separately [22,23,24,25]. Moreover, serological diagnosis (IFA and ELISA techniques) is used mainly in the case of chronically infected animals [20,23,24]. The effective treatment of EP, therefore, depends on the ability to differentiate between *T. equi* and *B. caballi* [26]. Thus, accurate and sensitive diagnostic methods that can differentiate between *T. equi, B*. *caballi,* and *T. haneyi* in animals that have mixed infections are required as a step toward implementing adequate control measures. To overcome the diagnostic drawbacks faced in Egypt, more sensitive and specific DNA amplification methods like PCR followed by sequencing of the amplicons could be used, especially in the prepatent phase infection of piroplasms [27].

Although uniplex (u) PCR assays are effective in the detection of single-species infection, they are time-consuming and expensive when applied on many samples that may have mixed infections [28]. The reverse line blot (RLB) assay has overcome this problem to a large extent by allowing the simultaneous detection of multiple parasite species in a single sample [29], but RLB requires expertise and specialized equipment, and the protocol is very labor-intensive [28]. Multiplex PCR (mPCR) is a single, lower cost, and technically less challenging approach that is able to amplify two or more target loci from one or more organisms using a mixture of specific primer pairs in a single reaction. Thus, mPCR could be a favorable tool for the diagnostic and epidemiological evaluation of TBD in endemic regions [28,30]. Therefore, the current work aimed to study the prevalence of EP using mPCR to detect *T. equi* and *B. caballi* simultaneously, targeting the 18S rRNA of both species. In addition, the current research addressed, for the first time, the detection of *T. haneyi* in Egypt through the examination of blood samples by conventional PCR (cPCR) followed by amplicon sequence comparison with South African and American *T. haneyi* isolates.

## 2. Results

### 2.1. Molecular Detection of Equine Piroplasmosis

#### 2.1.1. Multiplex PCR for the Simultaneous Detection of *T. equi* and *B. caballi*

**Figure 1:**
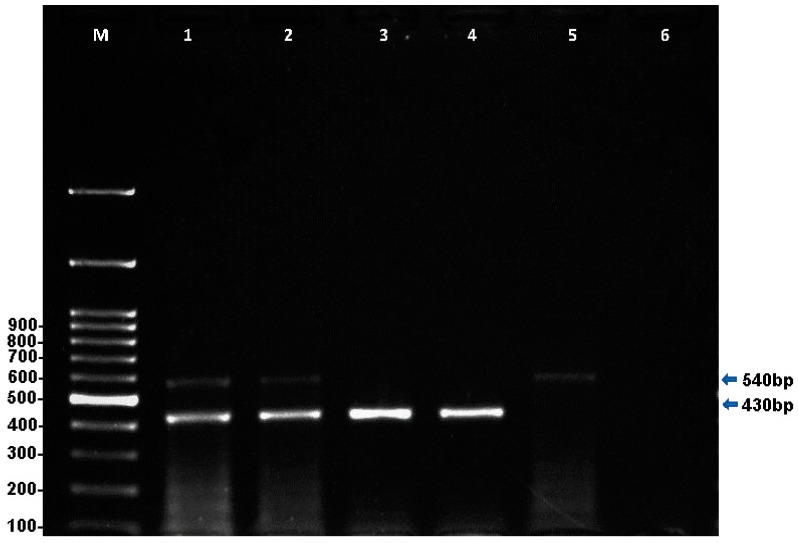
Multiplex PCR for the simultaneous detection of *T. equi* and *B. caballi* using a 2% agarose gel stained with SYBR safe; M: DNA ladder, lane 1: *T. equi* (430 bp) and *B. caballi* (540 bp) positive control DNA, lane 2: mixed infection with *T. equi* and *B. caballi*, lanes 3 and 4: sample infected with *T. equi*, lane 5: sample infected with *B. caballi*, and lane 6: negative control.

Multiplex PCR detected single *T. equi* infections in 26 (16.7%) (95% CI, 10.1–22.5%) equids, 16 (20.3%) (95% CI, 11.1–29.1%) horses, and 10 (13.1%) (95% CI, 5.5–20.6%) donkeys at the expected amplicon size of 430 bp (Figure 1). Similarly, a single infection with *B. caballi* was detected in one horse (1.2%) (95% CI, −0.1–3.6%) with an expected amplicon size of 540 bp (Figure 1). Co-infections with both parasites were found in two horses (2.5%) (95% CI, 0–5.9%) and one donkey (1.3%) (95% CI, 0.1–3.8%), with an overall prevalence of 1.9% (95% CI, 0–4.0) (three equines) (Table 1). Statistically, there was no significant difference in EP infection among horses and donkeys on the basis of mPCR data (*p* > 0.05); however, the difference between single *T. equi* and *B*. *caballi* infections was statistically significant (*p* < 0.05) as *T. equi* infection was more prevalent. 

#### 2.1.2. Conventional PCR Analysis for the Detection of *T. haneyi* in Egyptian Equids

**Figure 2:**
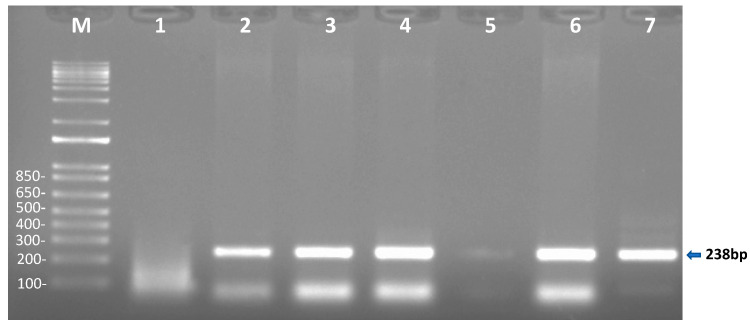
Conventional PCR of *T. haneyi* using a 1.5% agarose gel stained with SYBR safe; M: Ladder, lane 1: negative control, and lanes 2–7: *T. haneyi* positive amplicon.

*Theileria haneyi* was detected in 71 (45.8%) (95% CI, 37.4–53.6%) equids; 42 (53.1%) (95% CI, 40.4–62.1%) of these samples were derived from horses, and 29 (38.1%) (95% CI, 27.2–49.0%) from donkeys (Table 2). The positive samples gave the expected amplicon size of 238 bp (Figure 2). Statistically, there were no significant differences in infection with *T. haneyi* between horses and donkeys on the basis of the cPCR data (*p* > 0.05).

#### 2.1.3. Coinfections with *T. haneyi* (cPCR), *T. equi*, and *B. caballi* (mPCR)

The analysis of the mPCR and cPCR results of the 155 samples tested indicated that 3 horses and 18 donkeys were co-infected with both *T. haneyi* and *T. equi*. Additionally, co-infections with all three parasites (*T. equi*, *T. haneyi,* and *B. caballi*) were observed in two horses (95% CI, 0–5.9%) and one donkey (Table 2). Co-infections with *T. haneyi* and *B. caballi* were not observed.

### 2.2. Comparative Analysis and Sequence Conservation of the 18S rRNA Amplicons among Different Isolates

The 360-bp fragment of the *T. equi* 18S rRNA gene was amplified and sequenced from nine selected positive samples. The identity percent among the different Egyptian amplicons from *T. equi* and *B. caballi* is shown in supplementary Tables S1 and S2. Blast analysis indicated that the amplicon derived from the Egyptian isolates showed between 95.7 and 99% identity to previously published *T. equi* 18S rRNA gene sequences. In addition, the amplified *B. caballi* amplicon (540 bp) from two selected positive sample was sequenced. Blast analysis indicated that the *B. caballi* Egyptian isolate showed an identity percent ranging from 98.1 to 99.3% to published *B. caballi* isolates.

**Figure 3:**
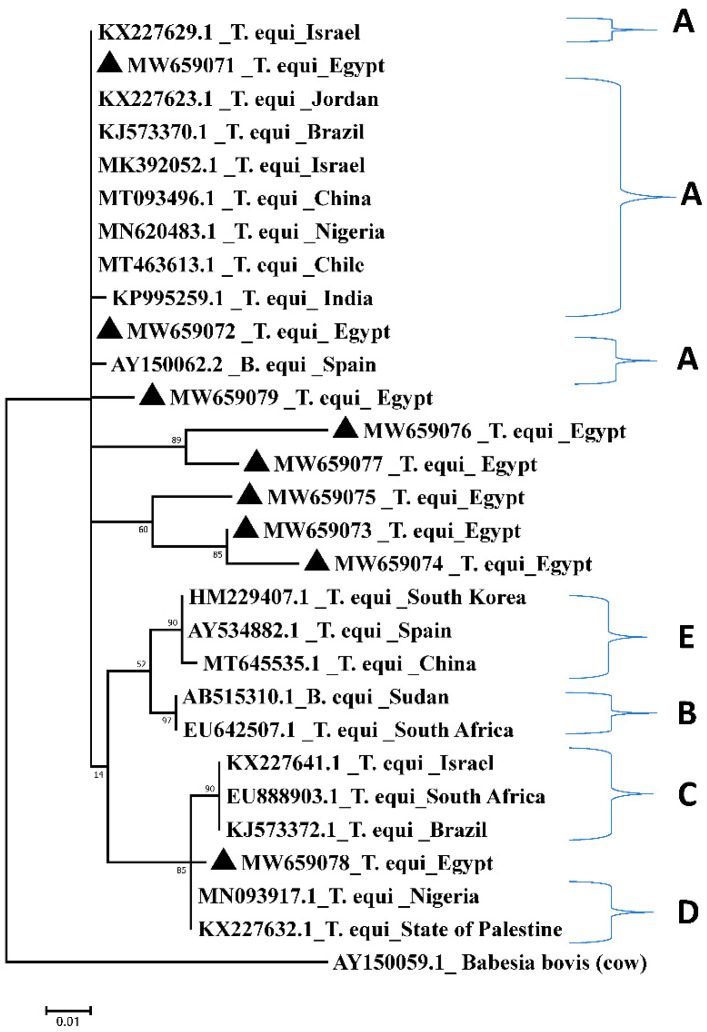
Comparative analysis by the Maximum Likelihood method of *T. equi* 18S rRNA gene. Egyptian isolates are labelled with a triangle. A, B, C, D, and E mean different genotypes. *Babesia bovis* AY150059 gene sequence was used as an outgroup.

Comparative analysis showed that one *T. equi* Egyptian amplicon derived from one horse with accession number MW659071.1 and two amplicons from donkeys with accession numbers MW659072.1 and MW659079.1 clustered with sequences from Chile (MT463613.1) [31], Israel (MK932052.1) [13], China (MT093496.1) [31], Jordan (KX227623.1) [32], and Nigeria (MN620483.1) [33], whereas only one Egyptian amplicon derived from one donkey (MW659078.1) clustered with sequences from the State of Palestine (KX227632.1) [32] and Nigeria (MN093917.1) [34]. In addition, three sequences derived from horses (MW659073.1, MW659074.1, and MW659075.1) and two from donkeys (MW659076.1 and MW659077.1) clustered together in a separate group from the other sequences obtained in the current study (Figure 3).

**Figure 4:**
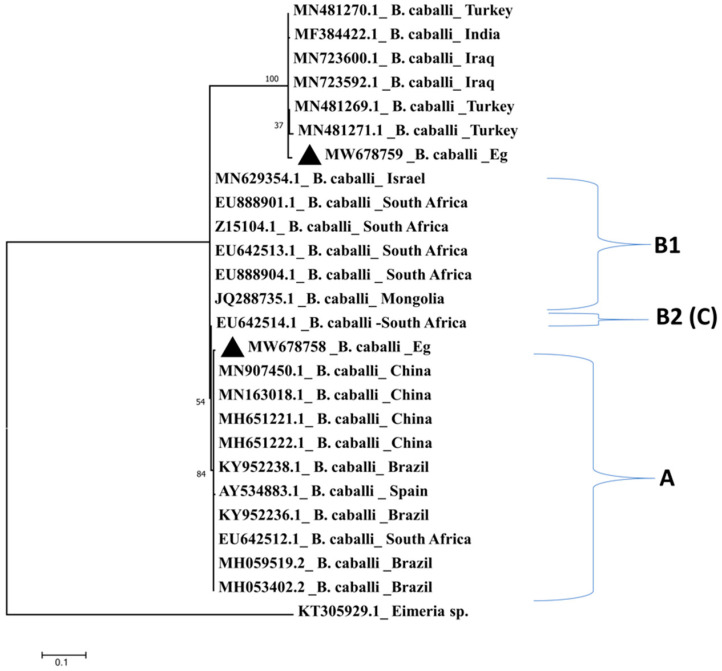
Comparative analysis by the Maximum Likelihood method of *B. caballi* 18S rRNA gene. Egyptian equine *B. caballi* isolates are labelled with a triangle. A, B1, and B2 (C) mean different *B*. *caballi* genotypes. *Eimeria* sp. KT305929 gene was used as an outgroup.

Similarly, the *B. caballi* Egyptian isolates showed 98.1–99.3% sequence identity with *B. caballi* sequences from China, Brazil, South Africa, Israel, Iraq, Turkey, and India. Comparative analysis of the *B. caballi* isolate (MW678758.1) from horses clustered with sequences from China (MN907451.1), Brazil (KY952238.1) [35], and South Africa (EU642512.1) [12], while a *B. caballi* isolate (MW678759.1) isolated from donkeys clustered in a separate clade with sequences from Iraq (MN723592.1), Turkey (MN481269.1), and India (MF384422.1) (Figure 4).

### 2.3. Sequencing Analysis of a T. haneyi Hypothetical-Protein-Coding Gene 

**Figure 5:**
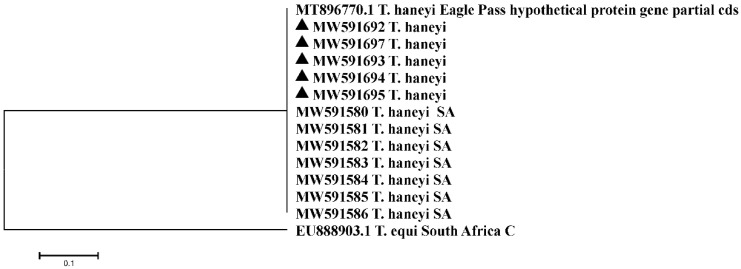
Comparative analysis by the Maximum Likelihood method of *T. haneyi* gene coding for a hypothetical protein. Egyptian samples are labelled with a black triangle. SA: South Africa *T. haneyi* isolates. *Theileria equi* genotype C of South Africa was used as the outgroup.

BLASTn analysis of the five *T. haneyi* Egyptian samples sequenced in this study showed 100% sequence identity to published *T. haneyi* sequences from South African isolates (MW591580-MW591586) [36] and to the published sequences of *T. haneyi* Eagle Pass strain gene for a hypothetical protein (MT896770.1) (Figure S1). The comparative analysis, based on amplicons derived from infected Egyptian horses (n = 2) (MW591694.1, MW591695.1) and donkeys (n = 3) (MW591692.1, MW591693.1, MW591697.1), indicated that the Egyptian *T. haneyi* sequences all clustered together with the reference *T. haneyi* sequence and with sequences from South African isolates; *T. equi* genotype C (18S r RNA) was selected as an outgroup (Figure 5).

## 3. Discussion

Piroplasms are Apicomplexa tick-borne parasites distributed worldwide which are responsible for piroplasmosis (theileriosis and babesiosis) in vertebrates. The aim of the present study was to use molecular methods for the detection of the prevalence of EP in Egypt caused by *T. equi* and *B. caballi.* We also aimed at detecting the occurrence of *T. haneyi* in equids in Egypt, which was unknown. Importantly, the DNA sequence data generated in this study also allowed for some genetic characterization of *T. equi*, *B. caballi*, and *T. haneyi* Egyptian strains currently circulating in this country.

The prevalence of *T. equi* was higher than that of *B. caballi*, and this is consistent with previous reports [37,38]. This phenomenon may be due to the increased susceptibility of *B. caballi* to treatment compared to *T. equi*. In addition, the horse immune system may be more efficient in eliminating *B. caballi*-infected erythrocytes than *T. equi*-infected ones, the latter parasites having a long persistence [9,39].

The result of this study also indicate that the prevalence of coinfections with both parasites (*T. equi* and *B. caballi*) in equids was 1.9%, which is lower than that detected in Mongolia (7.7%) [40] and Iraq (5.15%) [41] using mPCR and in Cuba (20%) [21] and Nigeria (2.7%) using nested PCR [9].

The observed difference in the prevalence of EP compared to other countries may be due to the type of equids (race or working) examined, hygienic measures, differences in environmental conditions—which can have a significant impact on tick activity—tick control strategies, number of samples analyzed, and type of PCR used for molecular diagnosis [24].

Blast analysis of the amplified fragments from *T. equi* and *B. caballi* showed sequence identities between 96 and 99% to published sequences. While lower sequence similarities may indicate distinct parasite species, it is important to note that the analysis was based on small fragments of the 18S rRNA gene. However, initial epidemiological studies on South African *T. equi* and *B. caballi* 18S rRNA gene sequences reported identities between 96.1 and 99.9% to the previously published *T. equi* sequence from South Africa (accession number: Z15105) and between 96.9 and 99.9% to a published *B. caballi* sequence from South Africa (accession number: Z15104). Phylogenetic analysis of the South African sequences and subsequently of sequences from other parts of the world led to the identification of distinct parasite genotypes, which may even represent distinct parasite species [36]. Therefore, the sequences obtained in this study could represent Egyptian isolates that belong to theses distinct parasite genotypes. However, amplification and sequencing of the complete 18S rRNA gene would be necessary to confirm these identities.

*Theileria haneyi* was defined as a new species infective to equids [5] and has since been reported to occur in several countries in North and South America, Africa, and Asia [5,9,36,42]. In the current study, *T. haneyi* was identified in both horses and donkeys in Egypt, and the sequence of the hypothetical-protein-coding gene was identical to the published *T. haneyi* Eagle Pass reference sequence and to sequences from South African isolates, confirming the presence of *T. haneyi* in Egypt, as reported here for the first time.

The results of the current study are in agreement with Sears et al., [10] who reported that coinfection of *T. haneyi* and *T. equi* could be induced experimentally in horses, which can explain the presence of the three parasites in naturally infected animals in our study. That means there was no cross immunity induced by *T. haneyi* and other two equine piroplasm (*T. equi* and *B. caballi*) and the infection with these two parasites does not protect equines from the infection with *T. haneyi* and vice versa.

The prevalence of *T. haneyi* either as single or as a mixed infection with *T. equi* and *B. caballi* was higher than that recorded for imported Argentine horses in Nigeria (2.7% and 0.6%, respectively) [9], and this observation may be explained by the factors mentioned earlier that include environmental conditions, husbandry, and tick vectors. Differences in sampling size and time of sample collection could also be contributing factors.

The application of new technologies with higher sensitivities and specificities could better facilitate the diagnosis of EP in Egypt. A multiplex EP real-time PCR assay targeting the 18S rRNA gene was developed for the simultaneous, quantitative detection of *T. equi* and *B. caballi* in field animals. Quantitative molecular genotyping assays for *T. equi* were also developed and enable the rapid detection of distinct *T. equi* parasite genotypes. Future studies in Egypt should focus on further characterizing the *T. equi* and *B. caballi* genotypes that may be circulating within the different governorates, with a view to determining risk factors in disease control. It has been noted that *T. haneyi* species classification was based on differences in the equi merozoite antigen (EMA) multigene family, and the identification of *T. haneyi* in South African horses infected with *T. equi* genotype C indicated that *T. haneyi* may be a subgroup of *T. equi* Genotype C [5,36]. The identification of *T. haneyi* in Egyptian equids is not surprising but warrants further investigation.

## 4. Materials and Methods

### 4.1. Collection of Field Samples

**Figure 6:**
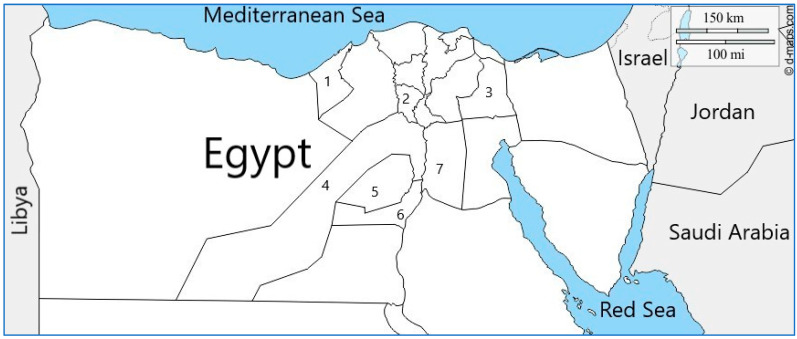
Geographic locations of the sampling sites in Egypt. 1: Alexandria, 2: Monufia, 3: Ismailia, 4: Giza, 5: Faiyum, 6: Beni Suef, and 7: Cairo.

Blood samples were collected from 155 apparently healthy equids (79 horses and 76 donkeys) from different governorates in Egypt (Cairo 30°2′0″ N, 31°14′0″ E, Giza 29°59′13.2″ N, 31°12′42.48″ E, Monufia 30°31′12″ N, 30°59′24″ E, Faiyum 29°18′30.14″ N, 30°50′38.78″ E, Beni Suef 29°4′0″ N, 31°5′0″ E, Ismailia 30°35′0″ N, 32°16′0″ E, and Alexandria 31°10′0″ N, 29°53′0″ E) (Figure 6).

The equid samples were collected from the following places: the Police Academy and Elzahraa-Stud in Cairo and the zoological garden Abattoir in Giza, National Research Centre veterinary caravans to Almonofia, Al fayoum, Beni Suef, Ismailia, and Alexandria governorates, Egypt. The blood samples were collected on EDTA-containing vials and transferred to the laboratory in ice boxes. Blood spots were prepared by applying 100 µL of blood on Whatman WB120410 FTA Elute Micro Card (GE Healthcare and Cytiva, North Bend, OH, USA). Ethical clearance for sample collection from equids was obtained through the Institutional Animal Care and Use Committee (IACUC) (Vet CU28/04/2021/297 and 28/04/2021).

### 4.2. DNA Extraction

Genomic DNA was extracted from FTA Elute Micro Card [43,44], following the manufacturer’s instructions.

Positive control DNA samples extracted from *T. equi* and *B. caballi* in vitro cultures were provided by the OIE equine piroplasmosis reference lab located in Pullman, WA, USA.

### 4.3. Molecular Detection of Equine Piroplasmosis by Three PCR Approaches

#### 4.3.1. Multiplex PCR (mPCR) for the Detection of *T. equi* and *B. caballi*

Field samples were tested for the presence of equine piroplasmosis using a published conventional mPCR assay designed for the simultaneous detection of *T. equi* and *B. caballi* infections [40]. The 18S rRNA gene was used, targeting the 943–1300-bp region for *T. equi* and the 562–1141-bp region for *B. caballi* [38,40]. Briefly, the universal forward primer Bec-UF2 and species-specific reverse primers (Cab-R, *B. caballi*; Equi-R, *T. equi*) were combined in reactions containing 3 µL of DNA sample, 12.5 µL of Sigma 2× JumpStart™ REDTaq^®^ ReadyMix™ (Foster City, California, USA), 5 μM of each primer, and 7.5 µL of nuclease-free water in a 25 µL total volume. Primers sequences are shown in Table 3. The amplification conditions were according to Abedi et al. [38], with minor modifications, which included an initial denaturation for 5 min at 94 °C, followed by 35 cycles each of 94 °C for 1 min as a denaturation period, an annealing period of 54 °C for 1 min, and an extension period at 72 °C for 1 min, with the addition of a final extension period of 7 min at 72 °C. The DNA extracted from *T. equi* and *B. caballi* in vitro cultures was used as a positive control, and the negative control was a no-template control (NTC). All amplicons were visualized by 2% agarose gel electrophoresis (Invitrogen, Waltham, USA).

#### 4.3.2. Uniplex PCR (uPCR) for Confirmation of the mPCR Results for the Detection of *T. equi* and *B. caballi*

Samples that tested positive for piroplasmosis using the mPCR assay were confirmed by performing uPCR assays. For the amplification of *T. equi* parasite DNA, the primers TBM and Equi-R were used, while the amplification of *B. caballi* was done using the primers Bec-UF2 and Cab-R (Table 3). The reactions were set up as previously described, and PCR amplification conditions were the same as those reported for the mPCR assay.

#### 4.3.3. Detection of *T. haneyi*

For the detection of *T. haneyi*, instead of performing a nested PCR as done by Knowles et al. [5], a gradient annealing temperature in PCR using the internal nested primers described in Table 3 was used. The best annealing temperature was 56 °C, which was chosen to complete the amplification process. Amplicons were visualized by 1.5% agarose gel electrophoresis.

### 4.4. Sequencing and Sequence Analysis

Samples (*T. equi* n = 9; *B. caballi* n = 2 and *T. haneyi* n = 5) that gave strong positive amplification reactions were selected for further sequencing and comparative analyses. Briefly, amplicons were purified using the GeneDirex PCR clean-up and Gel Extraction kit (Taiwan) according to the manufacturer’s instructions and sent for bi-directional sanger sequencing to Macrogen ( Seoul, South Korea ) using ABI3730XL DNA Sanger sequencer (ThermoFisher) (Waltham, MA, United States) All sequence data were edited using MEGA 7 software (https://www.megasoftware.net/download_form accessed on 2 January 2021). Query coverage and the percent of identity among the compared sequences were calculated by non-redundant National Centre for Biotechnology Information (NCBI) and Clustal Omega (https://blast.ncbi.nlm.nih.gov/Blast.cgi accessed on 2 January 2021) and (https://www.ebi.ac.uk/Tools/msa/clustalo/ accessed on 1 March 2021). In the present study, samples were aligned with the reference sequences for 18S rRNA representing *T. equi* (Z15105.1) [45] and for a gene coding a hypothetical protein of unknown function but specific for *T. haneyi* genome (MT896770.1 *T. haneyi* Eagle Pass strain) [5], available in the NCBI database. In addition, *B. caballi* gene sequence was kindly provided by Lowell S. Kappmeyer [Animal Diseases Research Unit, USDA-ARS, Pullman, WA 99164-6630, US]. Moreover, the *T. equi* and *B. caballi* sequences of the present study were compared with different 18S rRNA reference sequences collected from distinct geographical areas worldwide and available in GenBank (Tables S3 and S4) [46,47,48,49,50,51,52,53,54]. *T. haneyi* sequences were compared with the sequence of a hypothetical-protein-coding gene of *T. haneyi* Eagle Pass strain present in GenBank and with six *T. haneyi* South African (SA) isolate sequences [36]. All sequence data were edited using MEGA 7 software. Query cover and identity percentage among the compared sequences were calculated by NCBI and Clustal Omega (https://blast.ncbi.nlm.nih.gov/Blast.cgi accessed on 16 March 2021) and (https://www.ebi.ac.uk/Tools/msa/clustalo/ accessed on 23 February 2021). The resulted sequences data were submitted to GenBank to get accession numbers for *T. equi*, *B. caballi*, and *T. haneyi* Egyptian isolates.

### 4.5. Comparative Analysis

To assess the genetic diversity of hemoparasites within the study samples, species-specific dendrograms were constructed using a phylogenetic tree prediction generated by MEGA 7 (https://www.megasoftware.net/download_form accessed on 3 April 2021). This dendrogram was constructed using the Maximum Likelihood method based on the Kimura 2-parameter mode [55]. Egyptian *T. equi* and *B. caballi* isolates and the 18S rRNA gene of *T. equi* and *B. caballi* of different reference sequences in GenBank were used for comparative analyses, which were classified into genotypes A, B, C, D, and E for *T. equi* and genotypes A, B1, and B2 (C). The 18S rRNA gene sequences of *B. bovis* (AY150059.1) [56] were included in the dendrogram as outgroups for the *T. equi* dendrogram, while *Eimeria* sp. cytochrome oxidase subunit I (COI) gene (KT305929.1) [52] was used as the outgroup for the *B. caballi* dendrogram. Hypothetical-protein-coding gene of unknown function of *T. haneyi* Egyptian isolates, South African isolate (SA) [36], and *T.* haneyi Eagle Pass strain reference sequence [5] were used in *T. haneyi*’s dendrogram construction. *Theileria equi* genotype C South Africa (EU888903.1) [12] was used as the outgroup.

### 4.6. Statistical Analysis

The chi-square (χ^2^) test was applied at a probability of *p* < 0.05 to compare infection rates between equids determined by mPCR and cPCR. Significant associations were identified when a *p* value of less than 0.05 was observed [57].

## 5. Conclusions

The mPCR technique is a rapid diagnostic method for the simultaneous detection of both *T. equi* and *B. caballi*, especially in mixed-infected cases. This study represents a first report on the presence of *T. haneyi* in Egyptian equids and, specifically, in donkeys. Further investigations are required to determine the *T. equi* and *B. caballi* genotypes in Egypt and to study the impact of the presence of *T. haneyi* either as a single or as a co-infecting agent with other EP in disease control and how that can be involved in pathogen evolution.

## Figures and Tables

**Table 1:** Results of mPCR for the detection of *T. equi* and *B. caballi*.

Tested Animal	No.	Positive mPCR			
		EP	Single Infection		Co-Infection (*T. equi* and *B. caballi)*
			*T. equi*	*B. caballi*	
		No. (%, 95% CI)	No. (%, 95% CI)	No. (%, 95% CI)	No. (%, 95% CI)
Horses	79	19 (24.1%, 14.7–33.5%)	16 (20.3%, 11.1–29.1%)	1(1.2%,0.1–3.6)	2 (2.5%, 0–5.9%)
Donkeys	76	11 (14.4%, 6.5–22.2%)	10 (13.1%, 5.5–20.6%)	0	1 (1.3%, 0.1–3.8%)
Total equine	155	30 (19.3%, 13.1–25.5%)	26 (16.7%, 10.1–22.5%)	1(0.6%, 0–1.8%)	3 (1.9%, 0–4.0)

**Table 2:** Results for *T. haneyi* by cPCR and coinfection with *T. equi* and *B. caballi* in horses and donkeys determined by using mPCR.

Animal	No. of Tested Animals	Positive		
		*T. haneyi*	*T. haneyi* and *T. equi*	*T. haneyi, T. equi* and *B. caballi*
		No. (%, 95% CI)	No. (%, 95% CI)	No. (%, 95% CI)
Horses	79	42 (53.1%, 40.4–62.1%)	3 (4.5%, 0–9.0%)	2 (2.5%, 0–5.9%)
Donkeys	76	29 (38.1%, 27.2–49.0%)	18 (26.8%, 16.1–36.7%)	1 (1.3%, 0.1–3.8%)
Total equine	155	71 (45.8%, 37.3–53.6%)	21(13.5%, 8.8–18.8%)	3 (1.9%, 0–4.0%)

**Table 3:** Oligonucleotide primers used in molecular diagnosis.

Parasite	Primer Name	Gene Name	PCR Type	Amplicon Size	Primer Forward	Primer Reverse	Reference
*B. caballi*	*B. caballi* (diagnosis and sequencing)	18S rRNA	mPCR	540 bp	Bec-UF2 5-TCG AAG ACG ATC AGA TAC CGT CG-3	Cab-R 5-CTCGTTCATGATTTAGAATTG CT-3	[38,40]
*T. equi*	*T. equi* 1 (diagnosis)	18S rRNA	mPCR	430 bp		Equi-R 5-TGCCTTAAACTTCCTTGCGAT-3	
	*T. equi 2* (sequencing)	18SrRNA	uPCR	360 bp	TBM 5′-CTTCAGCACCTTGAGAGAAATC-3′	Equi-R 5′-TGCCTTAAACTTCCTTGCGAT-3	[14]
*T. haneyi*	*Th* int. (diagnosis and sequencing)	hypothetical protein gene of unknown function	cPCR	238 bp	Than_intfor 5′-GACAACAGAGAGGTGATT-3	Than_intrev 5′-CGTTGAATGTAATGGGAAC-3	[5]

## Data Availability

Not applicable.

## Supplementary Materials

The following are available online at https://www.mdpi.com/article/10.3390/pathogens10111414/s1, Table S1: Identity percent between *T. equi* Egyptian isolates analyzed in the present study. Table S2: Identity percent between *B. caballi* Egyptian isolates analyzed in the present study. Table S3: *T. equi* accession numbers of different 18s gene isolates used in dendrogram construction and their references. Table S4: *B. caballi* accession numbers of different 18s gene isolates used in dendrogram construction and their references. Figure S1: Alignment of the DNA sequences of five *T. haneyi* Egyptian (Eg) isolates (GenBank accession no. MW591692:MW591695 and MW591697) and six *T. haneyi* South African (SA) isolates of a hypothetical-protein-coding gene (GenBank accession number MW591580: MW591586) [BioEdit software].
