# Supplementary material for: Rapid Detection of Equine Piroplasms Using Multiplex PCR and First Genetic Characterization of Theileria haneyi in Egypt
Source: Pathogens. 2021 Oct 31;10(11):1414. doi: 10.3390/pathogens10111414 (PMC8620363; doi:10.3390/pathogens10111414)
Supplement: Supplementary file 1 [file pathogens-10-01414-s001.zip › pathogens-1416425-supplementary.pdf]

Supplementary Materials

Table S1. Identity percent between *T. equi* Egyptian isolates in the present study

	**MW659075**	**MW659073**	**MW659078**	**MW659074**	**MW659071**	**MW659072**	**MW659079**	**MW659077**
**MW659072**	96%	92%	96%	97%	100%	100%	98.5%	95%
**MW659079**	95.7%	91.6%	96.4%	95.5%	98.5%	98.5%	100%	94%
**MW659076**	93.3%	89.5%	91.8%	92.7%	92.7%	92.7%	92%	93%
**MW659077**	93%	88.5%	92.5%	93.5%	95%	95%	94%	100%
**MW659073**	93.7%	100%	91.6%	93%	91%	92%	91.6%	88.5%
**MW659078**	94%	91.6%	100%	93.4%	96%	96%	96.4%	92.5%
**MW659074**	94.8%	93%	93.4%	100%	97%	97%	95.5%	93.5 %

Table S2. Identity percent between *B. caballi* Egyptian isolates in the present study.

**Accession number**	**MW678758Eg**	**MW678759Eg**
**Identity percent**	76%	

Table S3. *T. equi* accession numbers of different 18s genes isolates that used in the phylogenetic tree construction and their references

Accession number	References
>MT645535.1	(Direct submission, NCBI, 2020)
>MT463613.1	[31]
>MN620483.1	[33]
>AY150059.1	[46]
>KP995259.1	[47]
>MK392052.1	[13]
>KJ573370.1	[48]
>KX227623.1	[32]
>AY150062.2	[46]
>KX227629.1	[32]
>AB515310.1	[49]
>EU642507.1	[12]
>KJ573372.1	[48]
>KX227641.1	[32]
>EU888903.1	[12]

>KX227632.1	[32]
>AB515307.1	[49]
>HM229407.1	[50]
>AY534882.1	[15,51]

Table S4: *B.caballi* accession numbers of different 18s genes isolates that used in the phylogenetic tree construction and their references.

Accession number	References
>KT305929.1	[52]
>MH651222	[16]
>EU888901.1	[12]
>Z15104.1_	[45]
>MN723592.1	(Direct submission, NCBI, 2019)
>MN481270.1	(Direct submission, NCBI, 2019)
>MF384422.1	(Direct submission, NCBI, 2017)
>MN481271	(Direct submission, NCBI, 2019)
>MN907450.1	(Direct submission, NCBI, 2020)
>MN163018.1	(Direct submission, NCBI, 2019)
>KY952236.1	[35]
>EU642512.1	[12]
>MN629354.1	[13]
>EU642513	[12]
>AY534883.1	[51]

>EU642514.1	[12]
>MH059519.2	[53]
>MH053402.2	[53]
>JQ288735.1	[54]

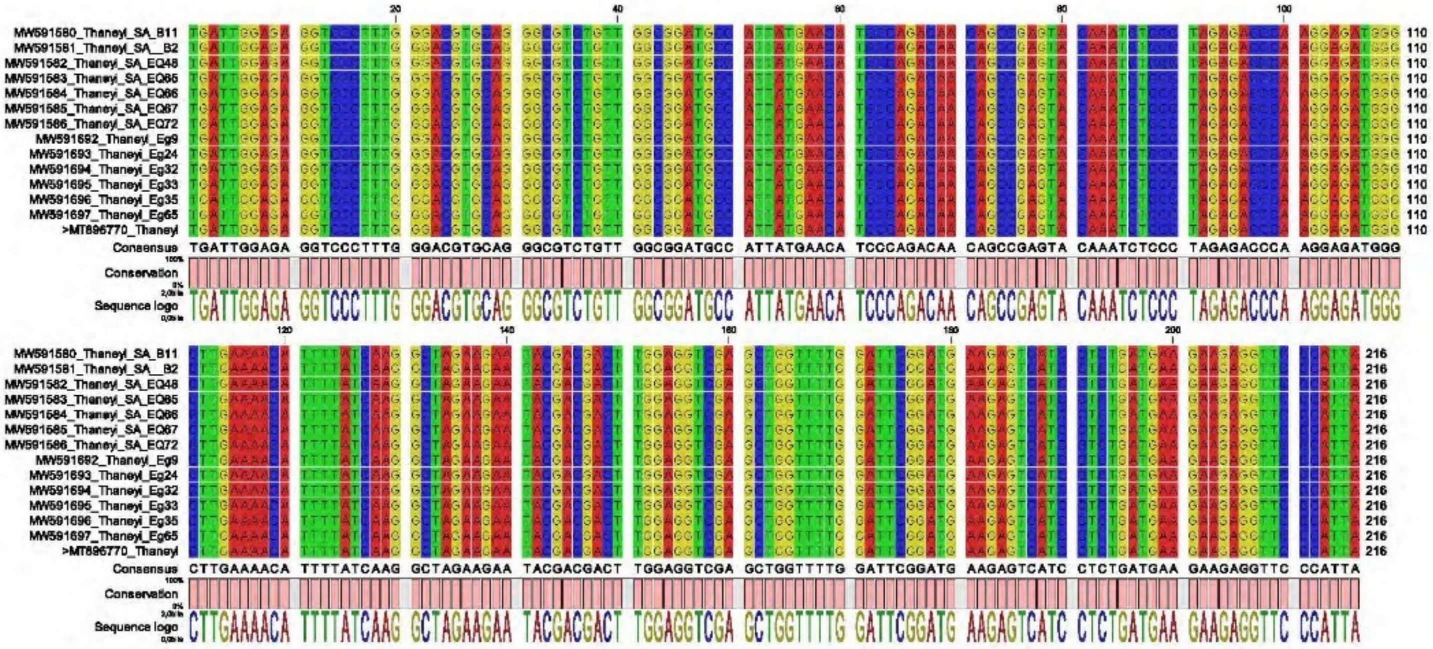

Figure S1. Alignment of the DNA sequences among five *T. haneyi* Egyptian (Eg) isolate (GenBank accession no. MW591692:MW591695 & MW591697) and the six *T. haneyi* South Africa (SA) isolate hypothetical protein gene (GenBank accession number MW591580: MW591586) [BioEdit software].
